# Erratum for Nikiforuk et al., “Performance of Immunoglobulin G Serology on Finger Prick Capillary Dried Blood Spot Samples to Detect a SARS-CoV-2 Antibody Response”

**DOI:** 10.1128/spectrum.03376-22

**Published:** 2022-10-10

**Authors:** Aidan M. Nikiforuk, Brynn McMillan, Sofia R. Bartlett, Ana Citlali Márquez, Tamara Pidduck, Jesse Kustra, David M. Goldfarb, Vilte Barakauskas, Graham Sinclair, David M. Patrick, Manish Sadarangani, Gina S. Ogilvie, Mel Krajden, Muhammad Morshed, Inna Sekirov, Agatha N. Jassem

**Affiliations:** a British Columbia Centre for Disease Control, Vancouver, British Columbia, Canada; b School of Population and Public Health, University of British Columbia, Vancouver, British Columbia, Canada; c Department of Pathology and Laboratory Medicine, University of British Columbia, Vancouver, British Columbia, Canada; d Department of Experimental Medicine, University of British Columbia, Vancouver, British Columbia, Canada; e Department of Pathology and Laboratory Medicine, British Columbia Children’s and Women’s Hospital, Vancouver, British Columbia, Canada; f Vaccine Evaluation Center, British Columbia Children’s Hospital Research Institute, Vancouver, British Columbia, Canada; g Women’s Health Research Institute, Vancouver, British Columbia, Canada; h Department of Pediatrics, British Columbia Children’s Hospital, Vancouver, British Columbia, Canada

## ERRATUM

Volume 10, no. 2, e01405-21, 2022, https://doi.org/10.1128/Spectrum01405-21.

Page 7, Table 2, column 5 (“Inclusion and exclusion criteria”), row 2: “≤18 yrs of age*^b^*” should read “≥19 yrs of age*^b^*”.

Page 7, Table 2, column 5 (“Inclusion and exclusion criteria”), row 3: “≥25 yrs of age*^b^*” should read “<25 yrs of age*^b^*”.

Page 7, Table 2, column 5 (“Inclusion and exclusion criteria”), row 4: “<25 or >69 yrs of age*^b^*” should read “>25 and <69 yrs of age*^b^*”.

Page 7, Table 2, column 5 (“Inclusion and exclusion criteria”), row 5: “PCR test negative for SARS-CoV-2 infection*^c^*” should read “Positive PCR test for SARS-CoV-2 infection*^b^*”.

